# Acute Effects of Particulate Air Pollution on Ischemic Stroke and Hemorrhagic Stroke Mortality

**DOI:** 10.3389/fneur.2018.00827

**Published:** 2018-10-02

**Authors:** Runhua Zhang, Gaifen Liu, Yong Jiang, Gang Li, Yuesong Pan, Yilong Wang, Zaihua Wei, Jing Wang, Yongjun Wang

**Affiliations:** ^1^Department of Neurology, Beijing Tiantan Hospital, Capital Medical University, Beijing, China; ^2^China National Clinical Research Center for Neurological Diseases, Beijing, China; ^3^Center of Stroke, Beijing Institute for Brain Disorders, Beijing, China; ^4^Beijing Key Laboratory of Translational Medicine for Cerebrovascular Disease, Beijing, China; ^5^Beijing Center for Diseases Prevention and Control, Beijing, China

**Keywords:** particulate matter, risk, stroke, ischemic, hemorrhagic, mortality

## Abstract

**Background and Purpose:** A large body of literature reported the association of particulate matter (PM) with stroke in high-income countries. Few studies have examined the association between PM and stroke in middle- and low-income countries and considered the types of stroke. In this study, we examined the short-term effects of particulate matter <2.5 μm in diameter (PM_2.5_) and particulate matter <10 μm in diameter (PM_10_) on ischemic stroke mortality and hemorrhagic stroke mortality in Beijing, China.

**Methods:** We used an ecological study design and quasi-Poisson generalized additive models to evaluate the association of PM_2.5_ and PM_10_ and cerebrovascular diseases mortality, as well as ischemic- and hemorrhagic stroke mortality. In the model, we controlled long-term and season trends, temperature, and relative humidity, the day of the week and air pollution. For cerebrovascular diseases mortality, we examined the effects stratified by sex and age with different lag days.

**Results:** A total of 48,122 deaths for cerebrovascular disease (32,799 deaths for ischemic stroke and 13,051 deaths for hemorrhagic stroke) were included in the study. PM_2.5_ was associated with stroke mortality. The 10 μg/m^3^ increase of PM_2.5_ was associated with the increase of mortality, 0.27% (95% CI, 0.12–0.43%) for cerebrovascular diseases, 0.23% (95% CI, 0.04–0.42%) for ischemic stroke and 0.37% (95% CI, 0.07–0.67%) for hemorrhagic stroke -. The associations between PM_10_ and mortality were also detected for cerebrovascular diseases and ischemic stroke, but not in hemorrhagic stroke. The stratified analysis suggested age and gender did not modify the effects of PM on mortality significantly.

**Conclusions:** Our study suggested that short-term exposure to ambient PM was associated with the risk of stroke mortality.

## Introduction

Globally, stroke is the second leading cause of premature mortality in 2015 (1). In the past two decades, age-standardized rates of stroke mortality have decreased; however, the absolute numbers have been increasing. It is estimated that there are 113 million disability-adjusted life-years (DALYs) due to stroke, 6.5 million deaths from stroke. Compared to the developed countries, there is an evident increase in DALYs and deaths in the developing countries (2). Due to higher stroke incidence and mortality rates, the burden of stroke is substantial in developing countries. According to a nationwide population-based survey, there are approximately 2.4 million new strokes, and 1.1 million people died from stroke annually in China (3). It is of significant public health interest to identify modifiable risk factors for stroke.

Apart from modifiable risk factors, air pollution has emerged as the third significant contributor to global stroke burden, accounting for 29.2% of the burden of stroke (4). One of the main priorities to reduce the stroke burden is to reduce exposure to air pollution, especially in low-income and middle-income countries (4). During the last two decades, numerous studies have performed to investigate associations between air pollution and daily admission or mortality (5–8). Besides gaseous pollutants, solid particles are an essential component of ambient air pollution. Recently, particulate matter (PM), especially ambient fine particulate matter air pollution (particulate matter with an aerodynamic diameter less than 2.5 μm, PM_2.5_), has received particular attention for its high toxicity. Identification of specific air pollutant which is a potential risk factor for stroke, is important for policy making, risk assessment and intervention takes. However, the epidemiologic findings on PM and the risk of stroke were inconsistent, from no significant associations to positive associations (9–12). Moreover, few studies conducted in developing countries and distinguished the effects of PM on types of stroke (9, 11, 13). It is still not clear whether the effect of PM on ischemic stroke is the same with hemorrhagic stroke (12, 14, 15).

In the present study, we conducted a time-series study to evaluate the association between PM and the mortality by stroke types and to determine potential effect modifiers of the relationship between of PM and stroke mortality.

## Materials and methods

### Study area and population

Beijing is the capital of China and located at 39°26–41°03 north latitude, 115°25–117°30 east longitude, covers 16411 km^2^. The population of Beijing registered residence was about 13 million during the study period (Jan 2014 to Dec 2016). The study area contains sixteen urban and suburban districts. Beijing has a temperate monsoon climate and features a four season. The primary air pollution source is automobile exhaust emissions, industrial emissions and wind-blown dust (16).

The study was approved by the ethics committee of Beijing Tiantan hospital. Because all of the data were de-identifier and the data were analyzed at the aggregate level, informed consent from the participant has been waived in this study.

### Stroke mortality data

The daily mortality counts of stroke from 1 January 2014 to 31 December 2016 were obtained from Beijing Center for Diseases Prevention and Control. At the time of death, death certificates are issued by community doctors for the deaths at home, or by hospital doctors for the deaths at hospitals (17). The death reasons were coded according to the International Classification of Diseases 10th version (ICD-10). I60-I69 were used for cerebrovascular disease, I63 and I63.9 for ischemic stroke, and I60-I62 and I69.1 for hemorrhagic stroke-. The Chinese Center for Disease Control and Prevention implemented strict quality control procedures to ensure of the accuracy and completeness of the death data (18). Moreover, in order to explore the susceptible populations, we divided mortality data -by age group (<45 years, 45–64 years and 65 years or older) and gender.

### Environmental data

We obtained daily air pollution data from January 2014 to December 2016, including PM_2.5_, PM <10 μm in aerodynamic diameter (PM_10_), nitrogen dioxide (NO_2_), sulfur dioxide (SO_2_), ozone (O_3_) from China Air Quality Online Analysis Platform (https://www.aqistudy.cn/). The data displayed on the platform was from the Beijing Environmental Protection Monitoring Center. There were 35 automated monitoring stations located in Beijing. In this study, except O_3_ that 8 h average concentration was used, for other air pollutants, we used 24 h average concentrations, which were calculated form all valid monitoring stations. At each monitoring station, the local government has mandated detailed quality assurance and quality control programs (17). In order to control the effect of weather conditions on stroke mortality, meteorological data containing daily mean temperature and relative humidity were obtained from the China Meteorological Data Sharing Service System (http://data.cma.cn/).

### Statistical analysis

We applied the generalized additive models with quasi-Poisson regression to estimate the associations between ambient PM_2.5_ and PM_10_ and the risk of stroke mortality, as previously described (19). In order to control the long-term and seasonal trends of daily stroke mortality, we used natural cubic spline with 8 degrees of freedom (df) per year in the model. The day of the week was introduced as an indicator variable in the model. Additionally, we - applied natural smooth spline with 3 df for temperature and humidity to control the confounding effects. PM_2.5_ or PM_10_ was incorporated in the established basic model to examine its effects on stroke mortality, as well as ischemic stroke and hemorrhagic stroke separately.

The main model structure is:

$$\begin{array}{l} \;In\left[E\left(Y_{t}\right)\right]=\beta_{0}+\beta_{1}PM+\beta_{2}DOW+s_{1}\left(temp,df=3\right)\\ +s_{2}\left(hum,df=3\right)+s_{3}\left(time,df=8/year\right), \end{array}$$

where *E*(*Y*_*t*_) is the expected stroke mortality count on day *t*, PM is particular matter, DOW is the day of the week, temp is the average temperature on the current day, hum is the relative humidity of the current day, time is calendar time, β is the regression coefficient and s indicates a smoothing spline. The results were summarized as percent change in daily mortality per 10 μg/m^3^ increase of PM. Furthermore, in order to examine whether gender and age modify the effect of PM on cerebrovascular deaths, - we evaluated the association stratified by gender and age. We tested the significance of subgroup differences through calculating$\left({\hat{Q}}_{1}-{\hat{Q}}_{2}\right)\pm 1.96\sqrt{S{Ê}_{1}+S{Ê}_{2}}$, where ${\hat{Q}}_{1}$and ${\hat{Q}}_{2}$ were estimates of the categories and *SÊ*_1_ and *SÊ*_2_ were their corresponding standard errors (20, 21).

In order to examine the stability of our results, we also performed sensitivity analyses. First, we fitted 2-pollutant models with adjustment for other air pollutants' (NO_2_, SO_2_, O_3_) effects respectively, to control the confounding effects of other pollutants. Second, different lag periods, containing single-day lags (from lag0 to lag2) and multiday lags (lag01 to -lag02) were used to investigate the lag patterns of air pollution. In single-day lags, a lag of 0 day (lag0) meant the concentration of current day and a lag of 1 day (lag1) referred to the concentration of the previous day. In multiday lags, lag01 corresponded to the average concentration of the current day and previous day, and lag02 referred to 3-day moving mean concentration of current day and previous 3 days (19). Additionally, we applied different df values for time trends to estimate the effects of air pollution.

All the analyses were conducted using the GAM procedure in SAS 9.4 (SAS Institute Inc, Cary, NC) and all reported P values are based on two-sided tests at the 0.05 level.

## Results

There were a total of 48,122 deaths of cerebrovascular disease from 1 January 2014 to 31 December 2016, including 32,799 ischemic strokes and −13,051 hemorrhagic strokes. The daily numbers of stroke deaths ranged from 11 to 76. The mean ages (SD) for cerebrovascular diseases, ischemic stroke and hemorrhagic stroke were 76.7 (11.6), 78.7 (10.0), and 71.7 (13.7), respectively. The percentage of male was 55.7% for cerebrovascular patients, 55.4% for ischemic strokes and 57.5% for hemorrhagic strokes. The mean daily average concentrations for PM_2.5_, PM_10_, NO_2_, SO_2_, and O_3_ were 79.1, 103.8, 50.6, 14.4, and 110.4 μg/m^3^, respectively. During the study period, the mean temperature was 13.8°C and relative humidity was 53.1% (Table 1).

**Table 1:** Distribution of Daily Stroke Mortality, Air Conditions and Air Pollution Variables.

	**Mean**	**Minimum**	**25%**	**50%**	**75%**	**Maximum**
Cerebrovascular disease, N per day	43.9	11.0	38.0	43.0	50.0	76.0
Ischemic stroke, N per day	29.9	10.0	25.0	30.0	34.0	55.0
Hemorrhagic stroke, N per day	11.9	1.0	9.0	12.0	14.0	24.0
PM_2.5_, μg/m^3^	79.1	5.2	29.8	60.0	106.4	477.5
PM_10_, μg/m^3^	103.8	1.7	47.0	86.8	135.9	480.8
NO_2_, μg/m^3^	50.6	8.1	33.5	44.5	61.4	153.5
SO_2_, μg/m^3^	14.4	1.8	3.6	7.9	17.4	133.1
O_3_, μg/m^3^	110.4	3.0	57.0	92.0	158.5	343.0
Temperature, °C	13.8	−14.3	2.8	15.6	24.0	32.6
Humidity, %	53.1	8.0	37.0	53.0	68.5	99.0

*PM_2.5_ indicates particulate matter <2.5 μm in aerodynamic diameter, PM_10_ indicates particulate matter <10 μm in aerodynamic diameter, NO_2_, nitrogen dioxide; SO_2_, sulfur dioxide; O_3_, ozone*.

In the single-pollutant model, we observed a significant association between daily cerebrovascular diseases mortality and PM_2.5_ and PM_10_. When we examined the effects by the type of stroke, the associations between PM_2.5_ and ischemic stroke and hemorrhagic stroke death were positive. However, exposure to PM_10_ was related to increase the risk of ischemic stroke but not hemorrhagic. Each 10 μg/m^3^ increase of PM_2.5_ was associated with the increases of mortality, 0.27% (95% CI, 0.12–0.43%) for cerebrovascular diseases, 0.23% (95% CI, 0.04–0.42%) for ischemic stroke and 0.37% (95% CI, 0.07–0.67%) for hemorrhagic stroke. However, each 10 μg/m^3^ increase of PM_10_ was associated with the increases of mortality for cerebrovascular diseases and ischemic stroke [0.19% (95% CI, 0.07–0.32%), 0.16% (95% CI, 0.01–0.32%), respectively], but not in hemorrhagic stroke [0.20% (95% CI, −0.04 to 0.44%)].

**Figure 1:**
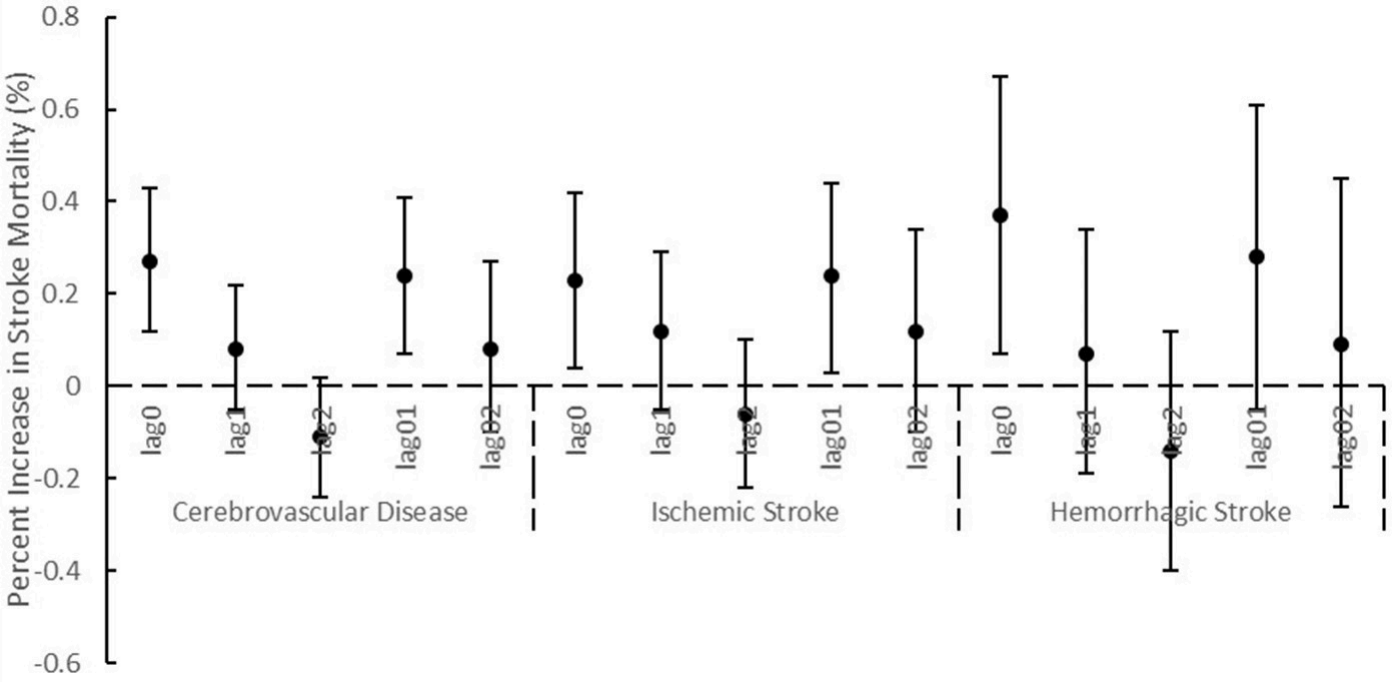
Percentage increase (mean and 95% confidence intervals) of stroke mortality associated with 10 μg/m^3^ increase of PM_2.5_ using different lag structures.

**Figure 2:**
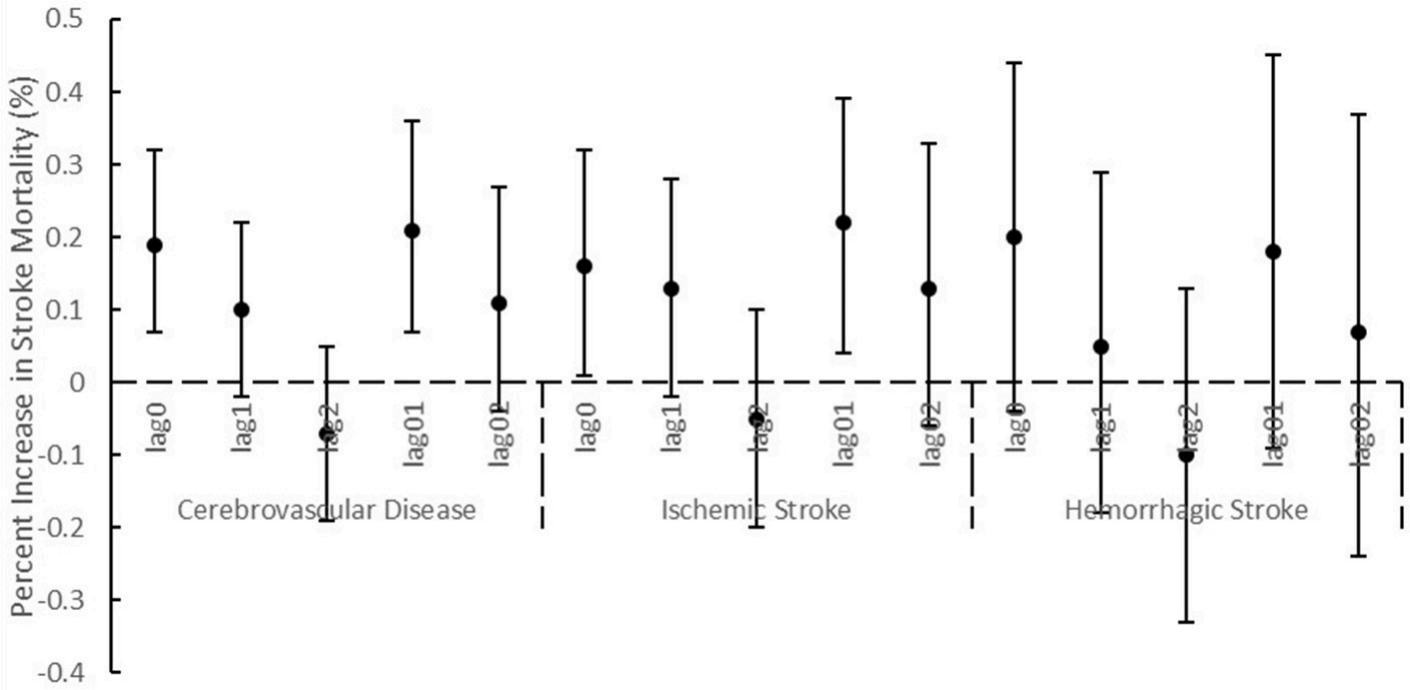
Percentage increase (mean and 95% confidence intervals) of stroke mortality associated with 10 μg/m^3^ increase of PM_10_ using different lag structures.

After we adjusted NO_2_ or O_3_ in the two-pollutant models, the associations between PM_2.5_ and cerebrovascular diseases remained statistically significant, and the consistent result was observed for ischemic stroke (Table 2). The association between PM_2.5_ and hemorrhagic stroke was attenuated after adding NO_2_ in the model. When NO_2_ and SO_2_ were adjusted, the associations of PM_10_ with cerebrovascular disease and ischemic stroke were not statistically significant. Using different lag periods, we found at lag 0, 1 and 0 to 1 days, the concentrations of PM_2.5_ were significantly associated with cerebrovascular diseases and ischemic stroke (Figure 1). A significant association of PM_10_ with cerebrovascular diseases and ischemic stroke was detected at lag 0 and 0 to 1 days (Figure 2). Results were essentially unchanged when the df for time trend was changed from 6 to 10 (data not shown).

**Table 2:** Percentage increase (mean and 95%confidence intervals) of stroke mortality associated with 10 μg/m^3^ increase of PM_2.5_ and PM_10_.

	**PM**_**2.5**_			**PM**_**10**_		
	**Mean**	**95% CI**	***P***	**Mean**	**95% CI**	***P***
Cerebrovascular Disease	0.27	0.12–0.43	0.0007	0.19	0.07–0.32	0.003
Adjusted SO_2_	0.18	0–0.37	0.0550	0.11	−0.04–0.26	0.1572
Adjusted NO_2_	0.28	0.05–0.51	0.0194	0.16	−0.03–0.35	0.1035
Adjusted O_3_	0.25	0.09–0.42	0.0020	0.18	0.06–0.31	0.0047
Ischemic Stroke	0.23	0.04–0.42	0.0191	0.16	0.01–0.32	0.0363
Adjusted SO_2_	0.10	−0.13–0.33	0.4056	0.05	−0.13–0.23	0.5666
Adjusted NO_2_	0.33	0.05–0.61	0.0228	0.22	−0.02–0.45	0.067
Adjusted O_3_	0.20	0.01–0.40	0.0396	0.16	0–0.31	0.0482
Hemorrhagic Stroke	0.37	0.07–0.67	0.0167	0.20	−0.04–0.44	0.1082
Adjusted SO_2_	0.46	0.10–0.82	0.0131	0.22	−0.06–0.51	0.1303
Adjusted NO_2_	0.29	−0.16–0.73	0.2083	0.03	−0.34–0.40	0.8732
Adjusted O_3_	0.35	0.04–0.66	0.0272	0.19	−0.05–0.44	0.1279

*PM_2.5_ indicates particulate matter <2.5 μm in aerodynamic diameter; PM_10_ indicates particulate matter <10 μm in aerodynamic diameter, NO_2_, nitrogen dioxide; SO_2_, sulfur dioxide, O_3_, ozone*.

The stratify analyzes by gender and age groups showed that female and the elder people were more likely to be vulnerable to PM_2.5_ and PM_10_. However, this did not hold at different lag days (Table 3).

**Table 3:** Percentage increase (mean and 95% confidence intervals) of stroke mortality associated with 10 μg/m^3^ increase of PM_2.5_ and PM_10_ using different lag structures by gender and age.

		**Gender**		**Age**		
		**Male**	**Female**	**~ <45**	**45~ <65**	**>=65**
PM_2.5_	lag0	0.24 (0.03–0.45)	0.32 (0.08–0.56)	0.25 (−1.03–1.54)	0.24 (−0.16–0.64)	0.28 (0.1–0.45)
	lag1	0.09 (−0.10–0.27)	0.08 (−0.13–0.29)	0.62 (−0.48–1.73)	−0.1 (−0.45–0.25)	0.11 (−0.04–0.26)
	lag2	−0.11 (−0.28–0.07)	−0.12 (−0.32–0.08)	0.44 (−0.62–1.51)	−0.38 (−0.72–0.03)	−0.06 (−0.2–0.09)
	lag01	0.22 (−0.01–0.45)	0.25 (0–0.51)	0.7 (−0.66–2.08)	0.05 (−0.38–0.48)	0.26 (0.07–0.45)
	lag02	0.08 (−0.17–0.32)	0.09 (−0.19–0.37)	0.86 (−0.6–2.34)	−0.26 (−0.72–0.21)	0.14 (−0.06–0.35)
PM_10_	lag0	0.14 (−0.03–0.31)	0.25 (0.06–0.44)	−0.46 (−1.5–0.58)	0.23 (−0.09–0.55)	0.19 (0.05–0.33)
	lag1	0.07 (−0.09–0.23)	0.14 (−0.04–0.32)	0.47 (−0.51–1.46)	−0.03 (−0.34–0.28)	0.12 (-0.02–0.25)
	lag2	−0.11 (−0.27–0.05)	−0.03 (−0.21–0.15)	0.38 (−0.59–1.35)	−0.41 (−0.72–0.1)	−0.01 (−0.14–0.13)
	lag01	0.15 (−0.04–0.35)	0.28 (0.07–0.5)	0.08 (−1.08–1.25)	0.13 (−0.23–0.49)	0.22 (0.07–0.38)
	lag02	0.04 (−0.17–0.25)	0.2 (−0.04–0.44)	0.35 (−0.92–1.65)	−0.2 (−0.6–0.2)	0.17 (0–0.35)

*PM_2.5_ indicates particulate matter <2.5 μm in aerodynamic diameter, PM_10_ indicates particulate matter <10 μm in aerodynamic diameter*.

## Discussion

To our knowledge, this is contemporary- study to analyze the association between PM_2.5_ and PM_10_ and the mortality by stroke types in the area with a high concentration of PM. Our present study suggested that short-term exposures to ambient PM_2.5_ and PM_10_ were associated with increased mortality of cerebrovascular diseases. When stratified by stroke types, we did not find evidence of an association between PM_10_ exposure and hemorrhagic stroke mortality.

We observed 0.27% (95% CI, 0.12–0.43%) and 0.19% (95% CI, 0.07–0.32%) increase in cerebrovascular diseases mortality with a 10 μg/m^3^ increase in PM_2.5_ and PM_10_, respectively. Due to large population exposure to ambient PM, it was of great benefits, though the effect was small. If the annual mass concentration of PM2.5 achieved the World Health Organization (WHO) Air Quality Guidelines (10 μg/m^3^) (22), about 400 stroke deaths will be avoided yearly in Beijing. The magnitude of the associations was lower than the results from a meta-analysis (1.1%, 95%CI 1.1–1.2% for each 10 μg/m^3^ increase of PM_2.5_; 0.3%, 95%CI 0.2 to 0.4% for each 10 μg/m^3^ increase of PM_10_) (23). The main reasons for this disparity may be that admission or mortality was to be recorded as an endpoint and most of the studies contained in this meta-analysis were from high-income countries. Additionally, the specific risks were volatile ranging from −2.9 to 31.4%. However, the the impact of PM on mortality in the present study was similar to that at another city in China (0.44%, 95%CI 0.16–0.72%) (24). This may occur due to the characteristic of air pollution, weather patterns and the economy. Hence, it is reasonable to summarize the effect according to geographical location.

Though ischemic stroke and hemorrhagic stroke share similar risk factors, they are different clinical entities (23, 25). In order to examine whether the effect of PM on different types of stroke was the same, ischemic stroke and hemorrhagic stroke was evaluated respectively. Our data showed that short-term elevations in PM_2.5_ increased the risk of death in both ischemic stroke and hemorrhagic stroke. Nevertheless, we found a significant association between ischemic stroke death and PM_10_ exposure, but not in hemorrhagic stroke. The studies of PM and hemorrhagic stroke were limited, and the outcomes were inconsistent (15, 26, 27). The mechanisms of hemorrhagic stroke and air pollution might be different from that of ischemic stroke and air pollution (23). Several mechanisms had been proposed, including exposure to particulate air pollution may induce inflammation (28), endothelial injury (29), atherosclerosis (30, 31), and can lower cerebral blood flow velocity (32).

There was not well documented referring to whether gender and age were effect modifiers. In the subgroup analysis, we did not find evidence that gender or age can modify the effect of PM on stroke. However, in some previous studies, it was suggested that females and the elderly were more likely to be vulnerable to air pollution (7, 33, 34). For instance, Kan et al. found that the effect of PM_10_ on total mortality among females was about twice those among males, though the difference was insignificant. Some stated that the gender difference could be partly explained by differences in particulate deposition, airway size (35), inflammatory response (36). Hong et al. found the elderly were more susceptible to PM_10_ (33). In the elderly, atherosclerosis is the main reason for ischemic stroke (37). Through pro-oxidant and pro-inflammatory effects, particulate matter modulates the progression of atherosclerosis, as a result of increasing the risk of ischemic stroke (38). However, the exact mechanisms of gender and age difference are unclear and deserve further investigation.

Several potential limitations of our study should be considered. First, as an ecological study, population level exposure was used, which may not reflect actual individual level exposure. The measurement error contains three components: the difference between an individual's deviation and the average personal exposure; the difference between the average personal exposure and the true ambient level; and the difference between the measured and the true ambient level. The first and third components are of the Berkson type and they are likely to have a small effect on the risk estimation (39). The effect of the second component may cause substantial bias, however, it tends to bias the results toward to null and underestimates the air pollutant effect (32, 40). Hence, the results should be interpreted with cautions and we cannot make causal inference form this study. Second, misclassification of cause of death may exist due to diagnostic or coding errors. However, the misclassifications seem to be unrelated with the air pollution levels and the errors may reduce the accuracy of the risk estimation (7). Third, because of data limitations, smoking-, drinking-, education- and complication-specific stroke mortality cannot be accessed, which prevented us from further exploring potential modifiers of association between air pollutants and ischemic stroke and hemorrhagic stroke.

In conclusion, PM exposure was associated with cerebrovascular death in Beijing, China. PM_2.5_ was associated with both ischemic stroke and hemorrhagic stroke death. However, short-term exposure to PM_10_ increased the risk of death in ischemic stroke but not in hemorrhagic stroke. Our study adds the evidence of the effect of PM on stroke in low-income countries, and it may have implications for public and environmental healthy policies.

## Author contributions

GL and YOW: study design; GL, JW, and ZW: data acquisition; YJ, YP, and RZ: data analysis and interpretation; RZ: drafting; GL, YIW, and YOW: revising and final approval.

### Conflict of interest statement

The authors declare that the research was conducted in the absence of any commercial or financial relationships that could be construed as a potential conflict of interest.
